# Modality and risk management for orthodontic extrusion procedures in interdisciplinary treatment for generating proper bone and tissue contours for the planned implant: a case report

**DOI:** 10.1186/s40729-015-0028-9

**Published:** 2015-10-23

**Authors:** Sachiko Maeda, Takeshi Sasaki

**Affiliations:** 1 Private practice limited to orthodontics, 8F Hankyu-Terminal-Bldg. 1-1-4 Shibata, Kitaku, Osaka 530-0012 Japan; 2 Private practice in general practice, 9F Nissei-Shinosaka-Bldg. 3-4-30 Miyahara, Yodogawa-ku, Osaka 532-0003 Japan

**Keywords:** Interdisciplinary treatment, Implant, Orthodontic extrusion (OE), Combination of orthodontic modalities, Risk management, Orthodontic extraction, Esthetics, Periodontal tissue health, Minimum intervention

## Abstract

In adult interdisciplinary treatments with using dental implants, limited orthodontic treatment, especially orthodontic extrusion (OE), offers many benefits by both correcting teeth alignment and by contributing to the regeneration of periodontal tissues. However, orthodontic procedures carry some risks and unpredictabilities that might compromise tooth and/or periodontal tissue health. Especially in complex cases, it is difficult to decide which orthodontic treatment modalities should be combined, in what sequences they should be applied, and what their force systems and treatment times are.

To achieve optimum results, some cases require two or more OEs to the same site being carried out at different times while taking the treatment effects into consideration. Such staged OE offers minimum intervention and maximum efficiency. In this case report, OE was first applied for orthodontic extraction. After bone regeneration followed by an implant placement and another surgical operation, a second OE was applied to align the inclination of an adjacent tooth. As a result, a predictable prognosis of implants as well as greatly improved esthetics and periodontal tissue health were achieved.

## Background

Adjunctive orthodontic treatment for adults is tooth movement that is carried out to facilitate other dental procedures necessary to control disease, restore function, and/or enhance appearance [1]. Orthodontic extrusion is an adjunctive orthodontic treatment with the potential to improve bone anatomy in consideration of implant placement [2].

Several studies have reported that orthodontic extrusion (OE) has many benefits in adult interdisciplinary treatment [3–6].

Because OE is regarded as being simpler and easier than comprehensive orthodontic treatment, this treatment can be mistakenly considered simple and completed without careful planning, which can lead to an unsatisfactory clinical outcome. Orthodontic treatments involve some risks for tooth and/or periodontal tissue that may lead to unpredictable and unsatisfactory results regardless of the complexity of treatments [7–9].

To achieve optimum results, it is important to decide which orthodontic treatment modalities should be combined. In addition, the sequence in which they are applied, the force system, and their treatment time must be considered [10].

In this case report, two LOTs were applied to the same site at different times, along with tooth extraction and guided bone regeneration followed by implant placement. For this patient, limited orthodontic treatment provided twice during the sequence of overall treatment resulting in improved esthetics and soft tissue health.

## Case presentation

A 28-year-old male visited our clinic with a chief complaint of poor esthetics in the maxillary anterior region. The patient was in good general health, and his medical and dental history indicated no contraindications to dental treatment.

**Fig. 1:**
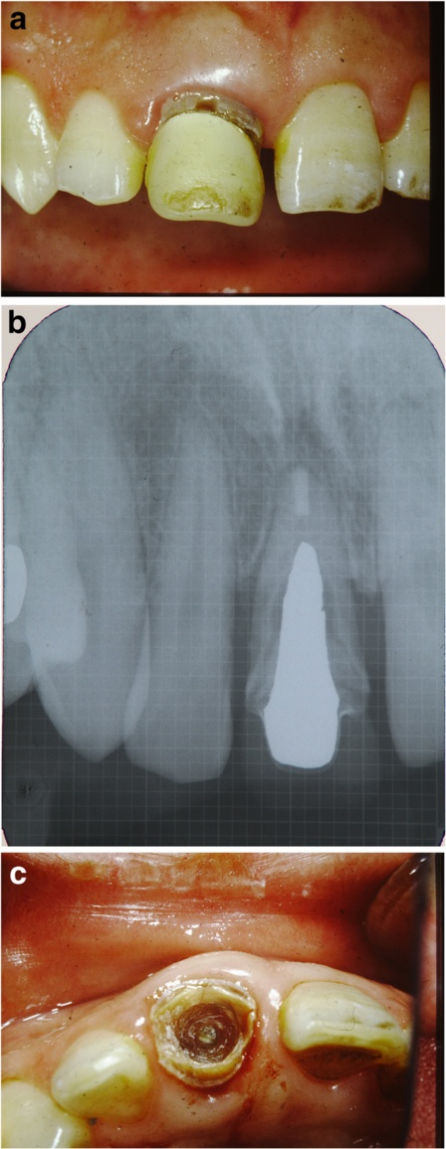
Initial clinical situation. **a** Flaring of the right maxillary incisors. **b** Initial dental radiograph showing short root, bone defect of the right maxillary incisor, and a fracture in the middle of the root. **c** After removal of the facial crown and metal post, the right maxillary incisor is seen to have large subgingival caries

The right maxillary central incisor had previously been restored with a porcelain veneer, while the right lateral incisor was inclined labially and distally. This resulted in spaces of 1.5 and 1.0 mm on the mesial and distal proximal area of the central incisor, respectively. Intraoral examination indicated that the right central incisor was elongated along with gingival recession. Radiographic examination revealed a large diameter metal post, bone resorption of up to one half of the resorbed short root, and a fracture in the middle of the root. Moderate bone resorption was also observed on the mesial aspect of the right lateral and left central maxillary teeth. Deep caries was found under the veneer and along the post space on the right central incisor (Fig. 1a–c).

The treatment plan consisted of the following items:

- Initial preparation with scaling and root planing
- Orthodontic extrusion and extraction of the right maxillary central incisor
- Implant placement with a bone grafting procedure
- A second implant operation for an abutment connection
- Orthodontic treatment for the right lateral incisor
- Final restoration and retention

**Fig. 2:**
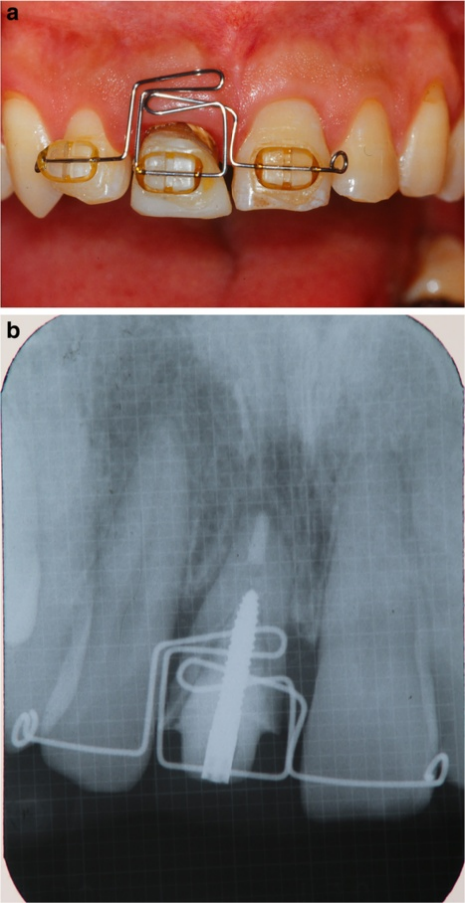
Initial orthodontic extrusion. **a** After the initial OE with the 016 NITI sectional arch wire, a 016 stainless steel wire with horizontal loops was placed to advance the extrusion. **b** Radiograph after OE

**Fig. 3:**
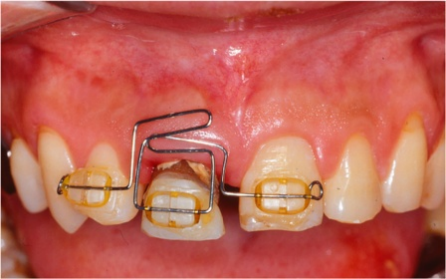
One month after the initiation of OE

**Fig. 4:**
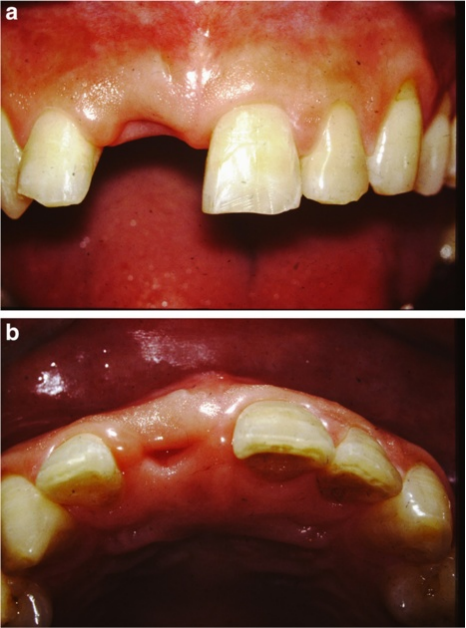
After the extraction of the maxillary central incisor (**a** frontal view, **b** occlusal view)

**Fig. 5:**
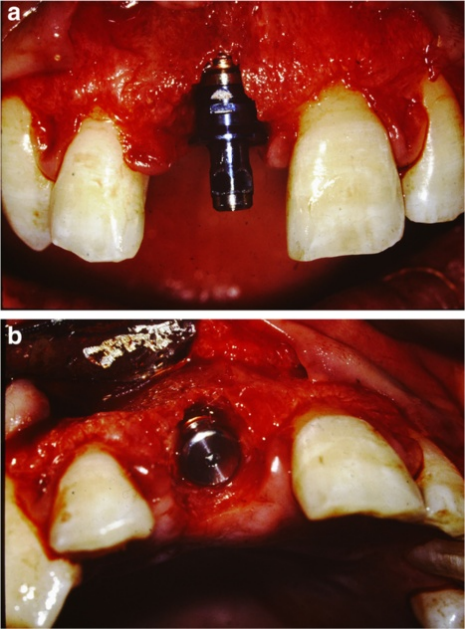
Placement of an implant and GBR (**a** frontal view, **b** occlusal view)

**Fig. 6:**
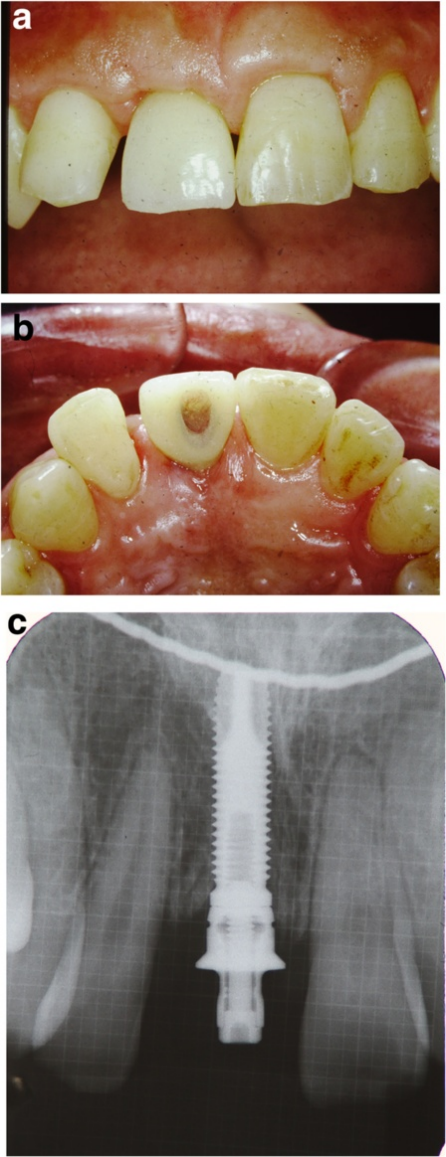
Provisional restoration on the implant (**a** frontal view, **b** occlusal view, **c** radiograph after placement of the provisional crown)

**Fig. 7:**
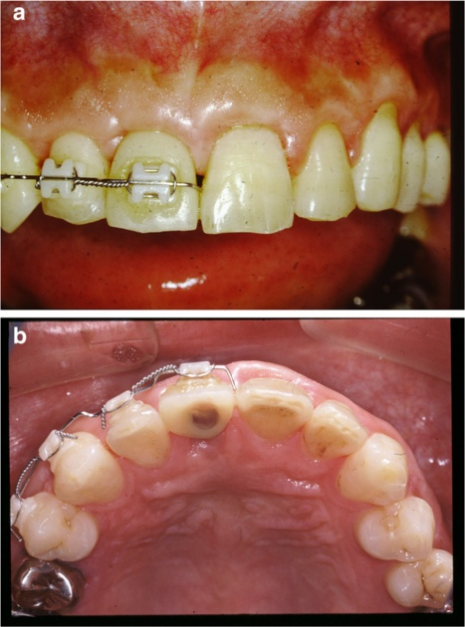
After the second orthodontic treatment to move the right maxillary lateral incisor in the mesio-lingual direction (**a** frontal view, **b** occlusal view)

**Fig. 8:**
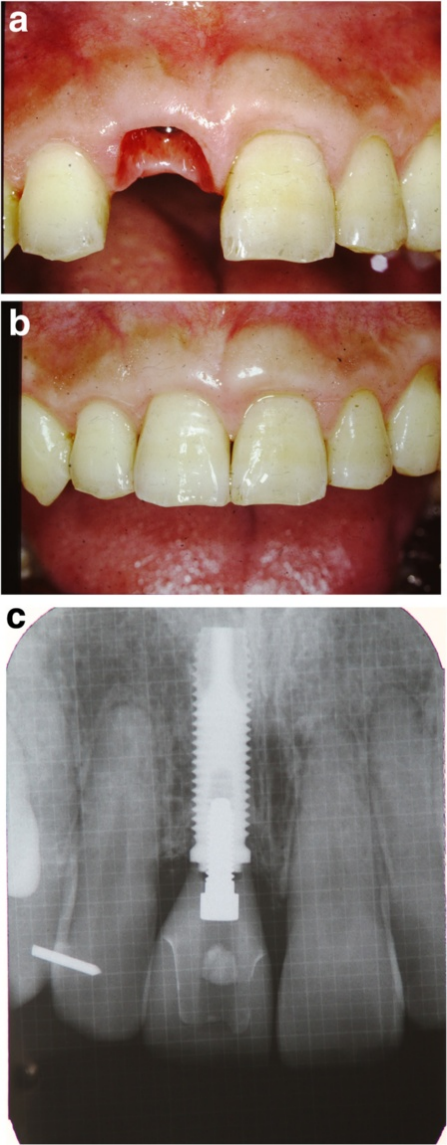
Definitive restoration. **a** Before definitive restoration on the implant cemented on the abutment. **b** Definitive restoration on the implant cemented on the abutment. **c** Radiograph of the definitive restoration

After removal of the existing restoration and the provisionalization of the right maxillary central incisor, scaling and root planing and open flap curettage were carried out. Brackets were then placed onto maxillary right lateral incisor, central incisor, and left central incisor (12, 11, 21 according to FDI system) and extrusion of 11 was completed by using a sectional arch wire with anchorage on #12, #21 with light force(30~50 g) in an incisal direction. Initially, a 016(0.016 mm diameter) nickel and titanium sectional wire and then a 016 stainless steel sectional wire with horizontal loop were used (Fig. 2a, b). Occlusal adjustments were made by grinding off the incisor area of the tooth. After 3 months, approximately 4 mm of tooth extrusion was achieved. (Fig. 3). The tooth was extracted 1 month after completing the extrusion (Fig. 4a, b). Six weeks after the extraction, a root form type implant (Osseotite Implant415, 3i )(4 × 15 mm) was placed into the site with tissue regeneration therapy using deproteinized cancellous bovine xenograft particles (Bio-Oss, Osteohealth) and enamel matrix derivative (Emdogain, Biora) and subepithelial connective tissue graft with dissolvable collagen membrane (Os-sx, Colbar) (10 × 10 mm) (Fig. 5a, b). Five months after the initial implant surgery, a second surgery for flap reflection was performed and provisional restoration was done with a temporary cylinder fixed on to the implant (Fig. 6a–c). A second orthodontic treatment to move the right maxillary lateral incisor in the mesio-palatal direction was initiated at this time by applying brackets on the right maxillary canine and provisional crown of the implant (Fig. 7a, b). Active tooth movement took 2.5 months, and 9 months retention was done with a wire-retainer cemented on the palatal side of the anterior teeth until a final implant crown was cemented on the abutment (Fig. 8a–c).

An esthetic implant-supported crown with symmetric soft tissue contours was achieved with the combined orthodontic extrusion, orthodontic alignment, and grafting procedures. The maintenance phase has been uneventful.

Orthodontic treatment plays a major role in adult interdisciplinary dentistry. In this case, orthodontic treatments were applied in two stages. In the first stage, OE of the right maxillary central incisor was carried out. In the second stage, the flaring adjacent lateral incisor on the same side was corrected. The first orthodontic treatment made optimal implant placement possible because of regenerated hard and soft tissue after the extraction. In addition, the first stage facilitated esthetic restoration with regenerated alveolar bone and soft tissue [11–13].

The second orthodontic treatment was employed to correct the position and angulation of the lateral incisor using an osseointegrated implant as the orthodontic anchor.

It was important that these orthodontic treatments were not applied simultaneously or with the same force system (orthodontic term: combination of all the forces and moments acting on these teeth).

There were two reasons for selecting a staged approach instead of a simultaneous one. First, if these tooth movements were attempted simultaneously, not only would the extrusion of the central incisor not be effectively achieved, but also the mesio-palatal movement of the right lateral incisor could not be sufficiently controlled. Since a lateral incisor will move to an unhealthy bone-defected area close to a central incisor, there could be the risk of an attachment loss of the lateral incisor [14–16]. In contrast, a staged approach would not incur the risk of attachment loss of the lateral incisor because regenerative therapy was applied first [17–19].

**Fig. 9:**
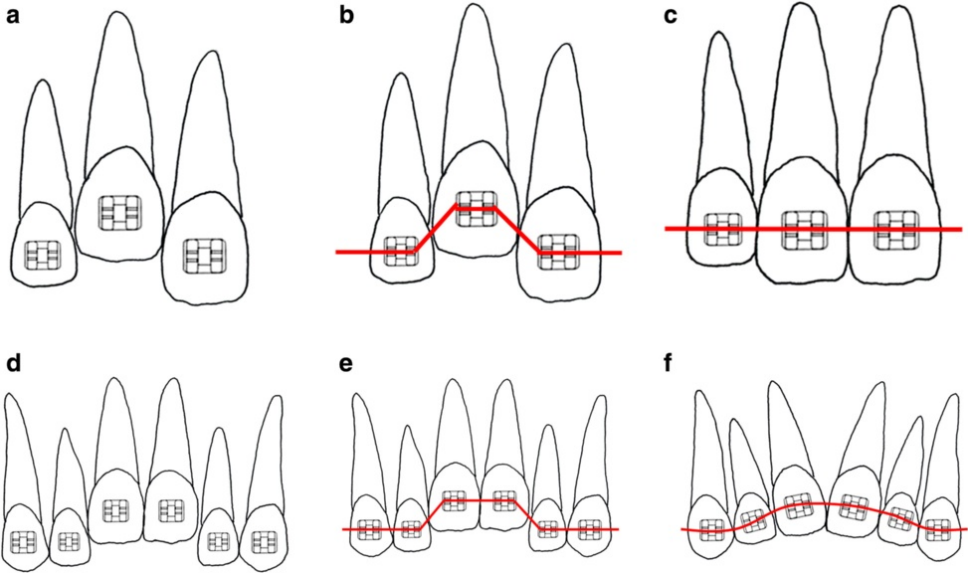
For extrusion of the right maxillary central incisor (**a**), the force system (**b**) would provide the desired result (**c**).For extrusion of the two maxillary central incisors (**d**), the force system (**e**) would provide inadequate results (**f**)

**Fig. 10:**
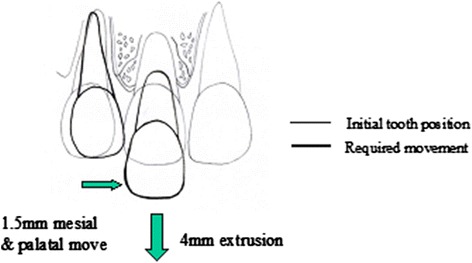
Required teeth movement (4 mm extrusion of the upper right central incisor and 1.5 mm mesial and palatal movement of the upper right lateral incisor). No force system with just one arch wire can facilitate this movement

Second, with an edgewise appliance, tooth movement can be controlled most efficiently when both adjacent teeth work as anchors [20] (Fig. 9a–c), and a symmetric counteractive orthodontic force can be applied between adjacent teeth (Fig. 9d–f). It is impossible to simultaneously perform a 4-mm extrusion of a central incisor (4 mm vertical movement) and a 1.5-mm mesio-palatal movement of a lateral incisor (1.5 mm lateral movement) (Fig. 10). Both an extrusion and a mesial movement of about 1 mm can be treated at the same time with one continuous arch wire using a leveling sequence [21]. However, it is not possible to move two teeth adjacent to each other in different directions and by different amounts using the same force system efficiently.

Orthodontic treatments in adults carry higher risks, such as gingival recession, alveolar bone resorption, and root resorption, compared with those in children [22, 23]. When a continuous arch wire is placed on many teeth to provide anchorage, balancing forces can result in unintended outcomes. In this case, the movement of the lateral incisor was achieved in a short period of time by using only one adjacent tooth as anchor because the anchorage for the implant was already in place. Adverse effects of orthodontic force could be minimized because the orthodontic treatment was performed in the shortest period of time possible and in a limited treatment area. The staged approach of orthodontic treatment in this study was carried out with minimum intervention and maximum efficiency.

## Conclusions

To prepare optimum implant placement site with improved esthetics and healthy periimplant tissue, orthodontic extrusion is one of the most effective and minimum invasive modalities, however, some cases require staged process with different type of orthodontic approach at different times.

## Consent

Written informed consent was obtained from the patient for the publication of this report and any accompanying images.
